# Synthesis and Anti-Tumor Activities of 4-Anilinoquinoline Derivatives

**DOI:** 10.3390/molecules21010021

**Published:** 2015-12-23

**Authors:** Dan Liu, Tian Luan, Jian Kong, Ying Zhang, Hai-Feng Wang

**Affiliations:** Department of Pharmaceutical Engineering, College of Parmaceutical and Biological Engineering, Shenyang University of Chemical Technology, Shenyang 110142, China; sapphire0614@163.com (T.L.); kongjianwm2006@163.com (J.K.); zhingky621@126.com (Y.Z.); 38whf@163.com (H.-F.W.)

**Keywords:** 4-anilinoquinolines, EGFR, antitumor, inhibitor

## Abstract

Twenty-two 7-fluoro (or 8-methoxy)-4-anilinoquinolines compounds were designed and synthesized as potentially potent and selective antitumor inhibitors. All the prepared compounds were evaluated for their *in vitro* antiproliferative activities against the HeLa and BGC823 cell lines. Ten compounds (**1a**–**g**; **2c**; **2e** and **2i**) exhibited excellent antitumor activity superior to that of gefitinib. Among the ten compounds; seven (**1a**–**c**; **1e**–**1g** and **2i**) displayed excellent selectivity for BGC823 cells. In particular; **1f** and **2i** exhibited potent cytotoxic activities against HeLa cells and BGC823 cells with better IC_50_ values than gefitinib.

## 1. Introduction

The epidermal growth factor receptor (EGFR) and its closely related family member HER2 play a critical role in mediating growth factor signaling, which make them interesting drug targets for oncology. EGFR and HER2 have been extensively investigated, and various classes of small molecule kinase inhibitors have emerged as promising strategies to inhibited EGFR and HER2 kinase activity.

**Figure 1:**
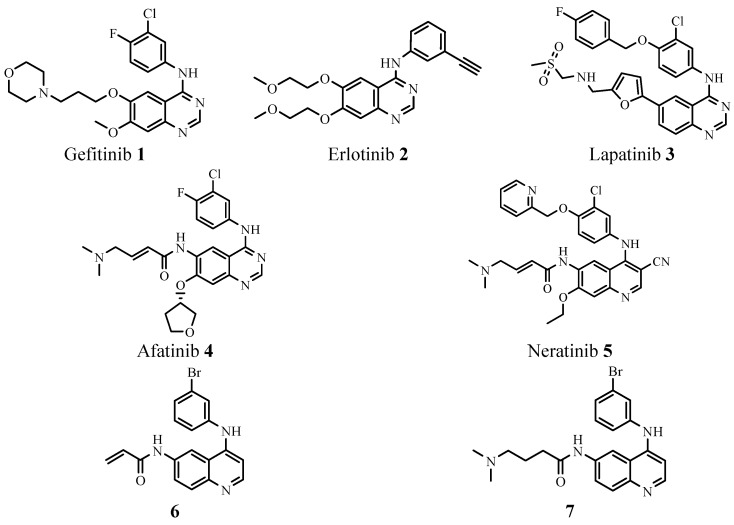
Several inhibitors of EGFR tyrosine kinases.

A considerable number of 4-anilinoquinazoline-based kinase inhibitors are known, including gefitinib (**1**), erlotinib (**2**) [1,2], lapatinib (**3**) [3,4], and afatinib (**4**) [5,6] (Figure 1). Previous SAR studies suggest that the quinazoline core was the best scaffold for the development of EGFR inhibitors and the quinazoline N3 interacts with the kinase domain via a water-mediated hydrogen bond to the side chain of the gatekeeper Thr790 of EGFR [7,8]. The quinazoline-based kinase inhibitors are widely used in medicinal chemistry and chemical biology research. For instance, Some C2 position modification of quinazoline-based compounds resulted in potent cytotoxic activities [9,10]. On the basis of the SAR studies of quinazolines, a series of compounds were developed where the N3 of the quinazoline was replaced by a C-CN group, such as neratinib (**5**, Figure 1) [11,12]. However, Rauh *et al.* have found that there was no evidence with the existence of a water molecule mediating the binding of N3 of the quinazoline core to the side chain of Thr790 by calculating the corresponding electron density maps. Quinolines **6** and **7** (Figure 1) were found to be highly active kinase inhibitors in biochemical assays and were further investigated for their biological effect on EGFR-dependent Ba/F3 cells and non-small cell lung cancer (NSCLC) cell lines [13]. Furthermore, studies have shown that the 4-anilino group can interact with the hydrophobic pocket of EGFR, the introduction of electron-donating groups in benzene ring of 4-anilinoquinazoline can increase the density of electron cloud on the quinazoline N1, and follow by increase the interaction with EGFR [14].

The aforementioned findings stimulated our interest in designing and synthesizing a series of 7-fluoro or 8-methoxy 4-anilinoquinolines which were acticipated to be as potent as their quinazoline counterparts. The activity of the target compounds were evaluated by human cervical cancer cell line (HeLa) and human gastric carcinoma cell line (BGC-823), and both of the cell lines had been proved to be highly expressed cell line of EGFR [15,16].

## 2. Results and Discussion

### 2.1. Chemistry

**Scheme 1:**
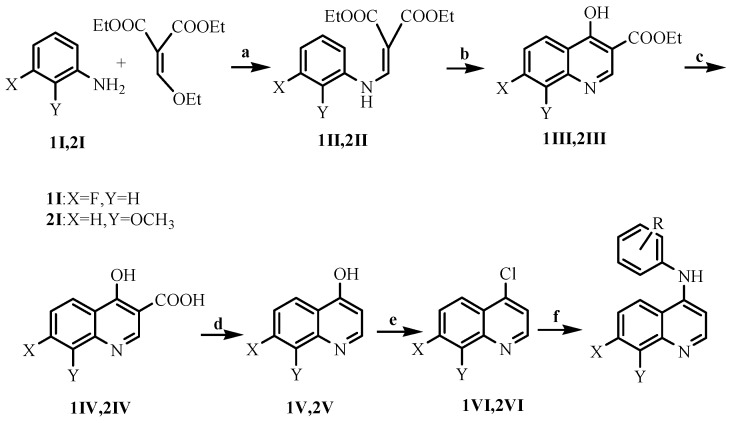
General procedure for the synthesis of target compounds **1a**–**h** and **2a**–**n**. *Reagents and conditions*: (**a**) EtOH, reflux; (**b**) Dowtherm A, 250–260 °C; (**c**) 10% NaOH, EtOH, reflux; (**d**) Dowtherm A, 240 °C; (**e**) POCl_3_; (**f**) *i*-PrOH, pyridine-HCl, substituted aniline, reflux.

The target compounds **1a**–**h** and **2a**–**n** were synthesized via a convenient six-step reaction depicted in Scheme 1 and the respective experimental details are given in Section 3.1. Twenty-two compounds were obtained and their MS, ^1^H-NMR spectroscopy data are provided in Section 3.1.

### 2.2. Biological Evaluation

The biological activities of all the target compounds **1a**–**h** and **2a**–**n** were evaluated *in vitro* by MTT assay against HeLa and BGC-823 cell lines with gefitinib (**1**) as the positive control. Their inhibition rate and IC_50_ values are listed in Table 1.

**Table 1:** Antiproliferactive activity of the target compounds on HeLa and BGC-823 cell lines. 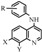

Compound	Substituents	Inhibition Rate % ^a^		IC_50_ (μM) ^b^	
		HeLa	BGC-823	HeLa	BGC-823
**1a**	X = F, Y = H, R = 3′-Cl	46.9	77.5	17.29	3.63
**1b**	X = F, Y = H, R = 4′-Cl	40.3	63.1	20.39	7.83
**1c**	X = F, Y = H, R = 3′-F	37.0	65.3	19.82	9.10
**1d**	X = F, Y = H, R = 4′-F	20.4	49.5	43.25	11.10
**1e**	X = F, Y = H, R = 3′-Cl,4′-Cl	40.8	56.9	36.69	8.29
**1f**	X = F, Y = H, R = 3′-Cl,4′-F	46.8	67.5	10.18	8.32
**1g**	X = F, Y = H, R = 4′-CH_3_	41.7	59.4	18.69	7.08
**1h**	X = F, Y = H, R = 4′-OCH_3_	36.1	35.1	55.76	21.61
**2a**	X = H, Y = OCH_3_, R = 3′-Cl	35.3	19.5	66.69	˃10
**2b**	X = H, Y = OCH_3_, R = 4′-Cl	31.5	13.1	˃10	˃10
**2c**	X = H, Y = OCH_3_, R = 3′-F	31.9	48.1	75.87	11.67
**2d**	X = H, Y = OCH_3_, R = 4′-F	18.9	37.5	˃10	46.41
**2e**	X = H, Y = OCH_3_, R = 3′-Cl,4′-Cl	30.8	48.7	45.87	11.45
**2f**	X = H, Y = OCH_3_, R = 3′-Cl,4′-F	37.2	20.2	45.84	˃10
**2g**	X = H, Y = OCH_3_, R = 4′-CH_3_	30.5	31.1	99.54	61.52
**2h**	X = H, Y = OCH_3_, R = 4′-OCH_3_	21.9	32.0	˃10	74.60
**2i**	X = H, Y = OCH_3_, R = 4′-CH(CH_3_)_2_	59.1	78.4	7.15	4.65
**2j**	X = H, Y = OCH_3_, R = 3′-Cl,4′-CH_3_	32.7	24.5	71.26	˃10
**2k**	X = H, Y = OCH_3_, R = 2′F, 3′-F, 4′-F	32.5	27.6	57.88	˃10
**2l**	X = H, Y = OCH_3_, R = 4′-NO_2_	35.8	36.8	13.48	˃10
**2m**	X = H, Y = OCH_3_, R = 4′-OH	32.7	25.6	39.58	˃10
**2n**	X = H, Y = OCH_3_, R = 3′-CN	28.6	16.0	30.61	˃10
Gefitinib		42.6	46.0	17.12	19.27

^a^ Inhibitory percentage of cells treated with each compound at a concentration of 10 μM for 96 h; ^b^ The agent concentration that inhibited HeLa and BGC-823 cells growth by 50%.

As shown in Table 1, 7-fluoro-4-anilinoquinolines **1a**–**g** displayed better cytotoxic activities against BGC-823 cells than HeLa cells and exhibited IC_50_ values within the 3.63–11.10 μmol/L range (on BGC-823 cells). It seems that substituent changes on the quinoline ring at the C7 or C8 carbons have a great influence on the antiproliferactive activity. In general, 7-fluoro-4-anilino-quinolines of these twenty-two compounds, were more active than those of the corresponding 8-methoxy-4-anilinoquinolines, as demonstrated by comparison of **1a**–**h** and **2a**–**h**. Thirdly, it was found that substituent changes on the benzene ring had little influence on antiprolifeactive activity, whether it was electron-donating or electron-withdrawing substituent at any position (**1a** *vs.* **1b** and **1d**; **2g** *vs.* **2b** and **2d**; **2g** and **2m** *vs.* **2l** and **2n**). Lastly, most of the 8-methoxy-4-anilinoquinolines exhibited moderate antiproliferactive activity against the HeLa and BGC-823 cell lines, except for compound **2i**. It was noteworthy that compound **2i** bearing **a** methoxy group on the quinoline ring and an isopropyl group on the benzene ring showed remarkable inhibitory effects on HeLa cells and BGC-823 cells **(**IC_50_ = 7.15 μM, IC_50_ = 4.65 μM), which represented a 2.4 and 4.1-fold increase in antitumor activity compared to gefitinib (IC_50_ = 17.12 μM against HeLa cells, IC_50_ = 19.27 μM against BGC-823 cells), respectively.

## 3. Experimental Section

### 3.1. Chemistry

All reagents and solvents were commercially available and were used without further purification. The melting points were determined on an electrically heated X-4 digital visual melting point apparatus (Tech, Beijing, China) and were uncorrected. ^1^H-NMR spectra were recorded on an ARX-300 or AV-600 spectrometer (Bruker, Fällanden, Switzerland) at room temperature, and chemical shifts were measured in ppm downfield from TMS as internal standard. Mass spectra were recorded on Thermo-Finnigan LCQ equipment (ThermoFinnigan, San Francisco, CA, USA) with the positive Electron Spray Ionization (ESI) mode and are reported as *m*/*z*. Analytical thin layer chromatography (TLC) on silica gel plates containing UV indicator (YuHua, Gongyi, China) was routinely employed to follow the course of reactions and to check the purity of products.

#### 3.1.1. Preparation of the Intermediates **1II**–**1VI**

Diethyl (ethoxymethylene) malonate (6.48 g, 30.00 mmol) and *m*-fluroaniline (2.78 g, 25.00 mmol) was stirred in refluxing ethanol (10 mL) for 1.5 h. The mixture was concentrated under reduced pressure. The residue was crystallized in petroleum ether, filtered off, and air-dried to get **1II**. Compound **1II** (2.80 g, 9.96 mmol) was stirred in Dowtherm-A (8 mL) for 0.5 h at 260 °C. After the reaction was over by TLC, the mixture was cooled to room temperature and petroleum ether was added to get the crude ester, which was further washed with petroleum ether to afford **1III**, as a white solid. **1III** was hydrolyzed in refluxing NaOH solution (10%, 20 mL) for 1.5 h to give **1IV**. Compound **1IV** (1.24 g, 6 mmol) was stirred in Dowtherm-A (10 mL) for 1 h at 240 °C. After the reaction was over as monitored by TLC, the reaction mixture was cooled to room temperature and the crude was washed with petroleum ether to afford **1V** as a white solid. To a solution of **1V** (1.20 g, 7.36 mmol) in 1,2-dichloroethane (30 mL), POCl_3_ (1.35 g, 8.83 mmol) was added dropwise. The mixture was refluxed for 1 h. Saturated NaHCO_3_ solution was added to neutralize the reaction mixture, which was worked up with 1,2-dichloroethane. The organic layer was dried over anhydrous MgSO_4_, filtered and concentrated *in vacuo*. The residue was purified by column chromatography (silica gel) using petroleum ether/ethyl acetate as an eluent (5:1) to produce **1VI** as a white solid [17]. **1VI**: ^1^H-NMR (300 MHz, CDCl_3_): 8.79–8.78 (d, 2-H, 1H), 8.27–8.24 (q, 5-H, 1H), 7.77–7.75 (q, 8-H, 1H), 7.47–7.46 (d, 3-H, 1H), 7.44–7.42 (q, 6-H, 1H), ESI-MS (*m*/*z*): 182.4[M + H]^+^.

#### 3.1.2. Preparation of the Intermediates **2II**–**2VI**

Compounds **2II**–**2VI** were prepared in the same manner as **2II**–**2VI**. **2VI**: ^1^H-NMR (300 MHz, CDCl_3_): 4.11 (3H, s, OCH_3_), 7.11 (1H, d, *J* = 7.2 Hz, Ar-H-7), 7.53–7.60 (2H, m, Ar-H-3, 6), 7.81 (1H, dd, *J* = 8.7 Hz, *J* = 1.2 Hz, Ar-H-5), 8.80 (1H, d, *J* = 4.8 Hz, Ar-H-2); ESI-MS (*m*/*z*):194.4[M + H]^+^.

#### 3.1.3. Preparation of the Title Compounds **1** and **2**

A mixture of compound **1VI** (0.040 g, 0.22 mmol), *m*-chloroaniline (0.036 g, 0.31 mmol) and pyridine hydrochloride was heated at reflux for 45 min in isopropanol (6 mL), after the reaction is over by TLC, it was cooled to room temperature and the petroleum ether (4 mL) and NaHCO_3_ (10 mL) were added into the reaction mixture. The product was filtered and recrystallised from ethanol to give the title compound **1**. Compound **2** was prepared in the same manner as **1**.

*4-(3′-Chlorophenylamino)-7-fluoroquinoline* (**1a**). Yield: 96.25%; white solid. m.p. 196–197 °C; ^1^H-NMR (600 MHz, CDCl_3_) δ: 8.62 (d, *J* = 4.8 Hz, 1H, ArH), 7.94 (dd, *J* = 9.0, 6.0 Hz, 1H, ArH ), 7.71 (dd, *J* = 4.2, 2.4 Hz, 1H, ArH), 7.37–7.27 (m, 3H, ArH), 7.19–7.17 (m, 2H, ArH), 7.01 (d, 1H, *J* = 5.4 Hz, ArH). ESI-MS (*m*/*z*): 273.4 [M + H]^+^.

*4-(4′-Chlorophenylamino)-7-fluoroquinoline* (**1b**). Yield: 92.93%. m.p. 194–195 °C, ^1^H-NMR (600 MHz, CDCl_3_) δ: 8.57 (s, 1H, ArH), 7.96 (dd, *J* = 8.4, 6.0 Hz, 1H, ArH), 7.70 (d, 1H, *J* = 10.2 Hz, ArH), 7.41–7.40 (m, 2H, ArH), 7.31 (t, *J* = 8.4 Hz, 1H, ArH), 7.27–7.25 (m, 2H, ArH), 6.90 (d, *J* = 4.8 Hz, 1H, ArH). ESI-MS (*m*/*z*): 273.4 [M + H]^+^.

*4-(3′-Fluorophenylamino)-7-fluoroquinoline* (**1c**). Yield: 95.37%; m.p. 173–174 °C; ^1^H-NMR (600 MHz, CDCl_3_) δ: 8.62 (s, 1H, ArH), 7.94 (dd, *J* = 9.0, 6.0 Hz, 1H, ArH), 7.70 (dd, *J* = 6.6, 2.1 Hz, 1H, ArH), 7.38 (dd, *J* = 14.7, 8.1 Hz, 1H, ArH), 7.34–7.31 (m, 1H, 1H, ArH), 7.06 (t, *J* = 4.5 Hz, 1H, ArH), 7.04–7.02 (m, 2H, ArH), 6.91–6.88 (m, 1H, ArH). ESI-MS (*m*/*z*): 257.4 [M + H]^+^.

*4-(4′-Fluorophenylamino)-7-fluoroquinoline* (**1d**). Yield: 93.66%; m.p. 178–179 °C; ^1^H-NMR (600 MHz, CDCl_3_) δ: 8.54–8.53(s, 1H, ArH), 7.94 (d, *J* = 6.0 Hz, 1H, ArH), 7.68 (dd, *J* = 10.2, 3.0 Hz, 1H, ArH), 7.32–7.28 (m, 3H, ArH), 7.17–7.13 (m, 2H, ArH), 6.74 (d, *J* = 5.4 Hz 1H, ArH). ESI-MS (*m*/*z*): 257.4 [M + H]^+^.

*4-(3′-Chloro-4′-chlorophenylamino)-7-fluoroquinoline* (**1e**). Yield: 91.34%; m.p. 191–192 °C; ^1^H-NMR (600 MHz, CDCl_3_) δ: 8.61 (d, *J* = 4.8 Hz, 1H, ArH), 7.97 (dd, *J* = 9.0, 6.0 Hz, 1H, ArH), 7.71 (dd, *J* = 9.6, 2.4 Hz, 1H, ArH), 7.48 (d, *J*=9.0Hz, 1H, ArH), 7.41 (d, *J* = 2.4 Hz, 1H, ArH), 7.34–7.27 (m, 1H, ArH), 7.16 (dd, *J* = 8.7, 2.7 Hz, 1H, ArH), 6.97 (dd, *J* = 5.4 Hz, 1H, ArH). ESI-MS (*m*/*z*): 307.3 [M + H]^+^.

*4-(3′-Chloro-4′-fluoroamino)-7-fluoroquinoline* (**1f**). Yield: 74.72%; m.p. 193–194 °C; ^1^H-NMR (600 MHz, CDCl_3_) δ: 8.58 (s, 1H, ArH), 7.93 (dd, *J* = 9.0, 5.4 Hz, 1H, ArH), 7.70 (dd, *J* = 10.2, 2.4 Hz, 1H, ArH), 7.38 (dd, *J* = 10.0, 2.4 Hz, 1H, ArH), 7.34–7.31 (m, 1H, ArH), 7.27–7.19 (m, 2H, ArH), 6.82 (d, *J* = 5.4 Hz, 1H, ArH). ESI-MS (*m*/*z*): 291.4 [M + H]^+^.

*4-(4′-Methylphenylamino)-7-fluoroquinoline* (**1g**)*.* Yield: 96.56%, m.p. 171–172 °C; ^1^H-NMR (600 MHz, CDCl_3_) δ: 8.50 (d, *J* = 5.4 Hz, 1H, ArH), 7.96 (dd, *J* = 9.3, 5.7 Hz, 1H, ArH), 7.68 (dd, *J* = 10.2, 2.4 Hz, 1H, ArH), 7.31–7.27 (m, 1H, ArH), 7.26–7.25 (m, 2H, ArH), 7.22–7.20 (m, 2H, ArH ), 6.83 (d, *J* = 4.2 Hz, 1H, ArH), 2.40 (s, 3H, CH_3_). ESI-MS (*m*/*z*): 253.4 [M + H]^+^.

*4-(4′-Methoxyphenylamino)-7-fluoroquinoline* (**1h**). Yield: 76.15%, m.p. 181–182 °C; ^1^H-NMR (600 MHz, CDCl_3_) δ: 8.46 (d, *J* = 5.4 Hz, 1H, ArH), 7.98 (dd, *J* = 9.0, 5.4 Hz, 1H, ArH), 7.68 (dd, *J* = 9.9, 2.7 Hz, 1H, ArH), 7.30–7.27 (m, 1H, ArH), 7.26–7.25 (m, 2H, ArH), 7.00–6.98 (m, 2H, ArH), 6.65 (d, *J* = 5.4 Hz, 1H, ArH). 3.88 (s, 3H, OCH_3_); ESI-MS (*m*/*z*): 269.3 [M + H]^+^.

*4-(3′-Chlorophenylamino)-8-methoxyquinoline* (**2a**). Yield: 76.16%, m.p. 250–251 °C; ^1^H-NMR (300 MHz, CDCl_3_) δ: 8.58 (d, *J* = 5.4 Hz, 1H, ArH), 7.45–7.50 (m, 2H, ArH), 7.40–7.30 (m, 1H, ArH), 7.14–7.10 (m, 2H, ArH), 7.06–6.98 (m, 2H, ArH), 6.88 (d, *J* = 5.4 Hz, 1H, ArH), 4.08 (s, 3H, OCH_3_); ESI-MS (*m*/*z*): 285.5 [M + H]^+^.

*4-(4′-Chlorophenylamino)-8-methoxyquinoline* (**2b**). Yield: 77.52%; m.p. 254–258 °C. ^1^H-NMR (300 MHz, CDCl_3_) δ: 8.60 (d, *J* = 5.1 Hz, 1H, ArH), 7.49–7.52 (m, 2H, ArH), 7.40–7.30 (m, 2H, ArH), 7.14–7.10 (m, 1H, ArH), 7.06–6.98 (m, 2H, ArH), 6.84 (d, *J* = 5.1 Hz, 1H, ArH), 4.08 (s, 3H, OCH_3_); ESI-MS (*m*/*z*): 285.5 [M + H]^+^.

*4-(3′-Fluorophenylamino)-8-methoxyquinoline* (**2c**). Yield: 75.06%; m.p. 267–269 °C. ^1^H-NMR (300 MHz, CDCl_3_) δ: 8.65 (s, 1H, ArH), 7.49–7.44 (m, 2H, ArH), 7.39–7.31 (m, 1H, ArH), 7.15 (dd, *J* = 5.1, 2.1 Hz, 1H, ArH), 7.08–6.99 (m, 3H, ArH), 6.88–6.82 (m, 1H, ArH), 4.08 (s, 3H, OCH_3_); ESI-MS (*m*/*z*): 269.3 [M + H]^+^.

*4-(4′-Fluorophenylamino)-8-methoxyquinoline* (**2d**). Yield: 84.59%; m.p. 256–258 °C. ^1^H-NMR (300 MHz, CDCl_3_) δ: 8.56 (d, *J* = 5.1 Hz, 1H, ArH), 7.52–7.41 (m, 2H, ArH), 7.30–7.27 (m, 2H, ArH), 7.15–7.05 (m, 3H, ArH), 6.82(d, *J* = 5.1 Hz, 1H, ArH), 4.08 (s, 3H, OCH_3_); ESI-MS (*m*/*z*): 269.3 [M + H]^+^.

*4-(3′-Chloro-4′-chlorophenylamino)-8-methoxyquinoline* (**2e**). Yield: 79.18%; m.p. 244–246 °C. ^1^H-NMR (300 MHz, CDCl_3_) δ: 8.63 (d, *J* = 5.1 Hz, 1H, ArH), 7.44–7.49 (m, 3H, ArH), 7.41 (d, *J* = 2.4 Hz, 1H, ArH), 7.16 (dd, *J* = 8.7, 2.4 Hz, 1H, ArH), 7.08 (d, *J* = 8.4 Hz, 1H, ArH), 7.03 (d, *J* = 5.1Hz, 1H, ArH), 4.08 (s, 3H, OCH_3_); ESI-MS (*m*/*z*): 319.3 [M + H]^+^.

*4-(3′-Chloro-4′-fluorophenylamino)-8-methoxyquinoline* (**2f**). Yield: 80.14%; m.p.247–249 °C. ^1^H-NMR (300 MHz, CDCl_3_) δ: 8.59 (d, *J* = 5.1 Hz, ArH), 7.38–7.36 (m, 1H, ArH), 7.20–7.17 (m, 2H, ArH), 7.07 (dd, *J* = 6.6, 2.4 Hz, 1H, ArH), 6.88 (d, *J* = 5.1 Hz, 1H, ArH), 4.08 (s, 3H, OCH_3_); ESI-MS (*m*/*z*): 303.3 [M + H]^+^.

*4-(4′-Methylphenylamino)-8-methoxyquinoline* (**2g**). Yield: 78.46%; m.p. 258–260 °C. ^1^H-NMR (300 MHz, CDCl_3_) δ: 8.56 (d, *J* = 5.4 Hz, 1H, ArH), 7.43–7.47 (m, 2H, ArH), 7.22–7.17 (m, 4H, ArH), 7.06 (dd, *J* = 6.9,1.5 Hz, 1H, ArH), 6.93 (d, *J* = 5.4 Hz, 1H, ArH), 4.08 (s, 3H, OCH_3_), 2.39 (s, 3H, CH_3_); ESI-MS (*m*/*z*): 265.4 [M + H]^+^.

*4-(3′-Methoxyphenylamino)-8-methoxyquinoline* (**2h**)*.* Yield: 80.96%; m.p. 231–233 °C. ^1^H-NMR (300 MHz, CDCl_3_) δ: 8.61 (d, *J* = 5.1 Hz, ArH), 7.49–7.41 (m, 2H, ArH), 7.32 (t, *J* = 5.4Hz, 1H, ArH), 7.13–7.10 (m, 1H, ArH), 7.06 (dd, *J* = 6.9, 1.8 Hz, ArH), 6.90–6.84 (m, 2H, ArH), 6.73 (dd, *J* = 8.1, 2.1 Hz, 1H, ArH), 4.08 (s, 3H, OCH_3_), 3.83 (s, 3H, OCH_3_); ESI-MS (*m*/*z*): 281.2 [M + H]^+^.

*4-(4′-Isopropylphenylamino)-8-methoxyquinoline* (**2i**). Yield: 79.47%; m.p. 236–238 °C. ^1^H-NMR (300 MHz, CDCl_3_) δ: 8.53 (d, *J* = 5.1 Hz, 1H, ArH), 7.48–7.40 (m, 2H, ArH), 7.30–7.21 (m, 4H, ArH), 7.05 (dd, *J* = 6.9, 2.1 Hz, 1H, ArH), 6.99–6.96 (m, 1H, ArH), 4.08 (s, 3H, OCH_3_), 2.99–2.90 (m, 1H, CH), 1.29 (d, *J* = 6.9Hz, 6H, CH_3_) ; ESI-MS (*m*/*z*): 293.6 [M + H]^+^.

*4-(3′-Chloro-4′-methylphenylamino)-8-methoxyquinoline* (**2j**). Yield: 76.48%; m.p. 270–272 °C. ^1^H-NMR (300 MHz, CDCl_3_) δ: 8.61 (d, *J* = 5.4 Hz, 1H, ArH), 7.47–7.41 (m, 2H, ArH), 7.31(d, *J* = 2.4 Hz, 1H, ArH), 7.25 (d, *J* = 7.8 Hz, 1H, ArH), 7.12–7.04 (m, 2H, ArH), 6.99 (d, *J* = 5.1 Hz, 1H, ArH), 4.08 (s, 3H, OCH_3_), 2.39 (s, 3H, CH_3_); ESI-MS (*m*/*z*): 299.3[M + H]^+^.

*4-(2,3,4-Trifluorophenylamino)-8-methoxyquinoline* (**2k**). Yield: 70.00%; m.p. 238–239 °C. ^1^H-NMR (300 MHz, CDCl_3_) δ: 8.61 (d, *J* = 5.1 Hz, 1H, ArH), 7.57–7.45 (m, 2H, ArH), 7.24–7.16 (m, 1H, ArH), 7.10–6.84 (m, 2H, ArH), 6.84 (d, *J* = 5.4 Hz, 1H, ArH), 4.10 (s, 3H, OCH_3_) ; ESI-MS (*m*/*z*): 305.2 [M + H]^+^.

*4-(4′-Nitrophenylamino)-8-methoxyquinoline* (**2l**). Yield: 78.18%; m.p. 265–267 °C. ^1^H-NMR (300 MHz, CDCl_3_) δ: 8.79 (d, *J* = 4.8 Hz, 1H, ArH), 8.25 (d, *J* = 9 Hz, 2H, ArH), 7.51–7.46 (m, 2H, ArH), 7.37 (d, *J* = 4.8 Hz, 1H, ArH), 7.29–7.26 (m, 2H, ArH), 7.11 (t, *J* = 4.2 Hz, 1H, ArH), 4.10 (s, 3H, OCH_3_); ESI-MS (*m*/*z*): 296.2 [M + H]^+^.

*4-(4′-Hydroxyphenylamino)-8-methoxyquinoline* (**2m**). Yield: 58.17%; m.p. 236–238 °C. ^1^H-NMR (300 MHz, DMSO) δ: 9.44 (s, 1H, OH), 8.61 (s, 1H, ArH), 8.29 (d, *J* = 5.1 Hz, 1H, ArH), 7.89 (d, *J* = 8.7 Hz, 1H, ArH), 7.38 (t, *J* = 8.4 Hz, 1H, ArH), 7.12 (t, *J* = 8.4 Hz, 2H, ArH), 6.84 (d, *J* = 8.7 Hz, 2H, ArH), 6.56 (d, *J* = 5.1 Hz, 1H, ArH), 3.91 (s, 3H, OCH_3_); ESI-MS (*m*/*z*): 267.3 [M + H]^+^.

*4-(3′-Cyanophenylamino)-8-methoxyquinoline* (**2n**). Yield: 91.43%; m.p. 255–256 °C. ^1^H-NMR (300 MHz, CDCl_3_) δ: 8.68 (d, *J* = 5.1 Hz, 1H, ArH), 7.55–7.40 (m, 5H, ArH), 7.11–7.08 (m, 2H, ArH), 6.74 (s, 1H, ArH), 4.10 (s, 3H, OCH_3_); ESI-MS (*m*/*z*): 276.2 [M + H]^+^.

### 3.2. Cell Proliferative Assay

The antiproliferative activities of the prepared 4-anilinoquinolines against HeLa and BGC823 cell lines were evaluated by MTT assay *in vitro,* with gefitinib as the positive control. The negative control contains cells, culture medium, MTT and DMSO. All human tumor cells were cultured in RPMI 1640 medium supplemented with 10% fetal bovine serum (FBS). Cells were detached by trypsinisation, seeded at 1.0–2.0 × 10^3^ cells each well in a 96-well plate and incubated in 5% CO_2_ at 37 °C overnight, then treated with the test compounds at different concentration and incubated for 96 h. Fresh MTT solution was added to each well and incubated at 37 °C for 4 h. The MTT-formazan formed by metabolically viable cells was dissolved in 150 μL DMSO each well, and monitored by a microplate reader at dual-wavelength of 490 nm; IC_50_ was defined as the drug concentrations that inhibited the cell number to 50% after 96 h. Each test was performed three times.

## 4. Conclusions

In summary, two novel series of 4-anilinoquinolines were designed and synthesized as potentially potent and selective antitumor inhibitors. All of the final compounds were generated from aniline derivatives via six step reaction sequences including nucleophilic substitution, cyclization, hydroxylation, decarboxylation, chlorination and nucleophilic substitution. Among the 7-fluoro-4-anilinoquinolines, all the prepared compounds displayed some cytotoxic activity against the HeLa and BGC823 cell lines. Compounds **1a**–**g**, **2c** and **2e** displayed superior cytotoxic activities against the BGC823 cell line than gefitinib. Furthermore, compound **1f** displayed good cytotoxic activities against HeLa and BGC823 cells (IC_50_ value of 10.18 μM and 8.32 μM against HeLa and BGC823 cells, respectively). In particular, compound **2i** exhibited the most potent inhibitory activity against BGC823 cells (IC_50_ value of 7.15 μM and 4.65 μM against HeLa and BGC823 cells, respectively). The drug-like structural optimization based on the 4-anilinoquinoline skeleton will be reported in the future.
